# Efficacy and safety of avalglucosidase alfa in Japanese patients with late-onset and infantile-onset Pompe diseases: A case series from clinical trials

**DOI:** 10.1016/j.ymgmr.2024.101163

**Published:** 2024-12-27

**Authors:** Madoka Mori-Yoshimura, Hirotaka Ohki, Hideaki Mashimo, Kenji Inoue, Satoko Kumada, Takashi Kiyono, Akihiro Arimori, Mitsunobu Ikeda, Hirofumi Komaki

**Affiliations:** a Department of Neurology, National Center Hospital, National Center of Neurology and Psychiatry, Tokyo, Japan; b Department of Cardiology, Tokyo Metropolitan Children's Medical Center, Tokyo, Japan; c Department of Neuropediatrics, Tokyo Metropolitan Neurological Hospital, Tokyo, Japan; d Department of Neuropediatrics, Chiba Rehabilitation Center, Chiba, Japan; e Sanofi K.K., Tokyo, Japan; f Translational Medical Center, National Center of Neurology and Psychiatry, Tokyo, Japan

**Keywords:** Avalglucosidase alfa, Case series, Enzyme replacement therapy, Japanese, Infantile-onset Pompe disease, Late-onset Pompe disease

## Abstract

**Background:**

The efficacy and safety of avalglucosidase alfa for Pompe disease (PD) have been demonstrated in a global Phase 3 trial (COMET) in patients with late-onset PD (LOPD) and a global Phase 2 trial (Mini-COMET) in patients with infantile-onset PD (IOPD). This case series examines the individual results of three Japanese patients enrolled in these trials.

**Methods:**

Case reports were assembled from data collected in the COMET and Mini-COMET trials. Detailed methods have been reported previously. The primary endpoint of COMET was change from baseline to week 49 in upright forced vital capacity percent (FVC %) predicted. The primary endpoint of Mini-COMET was safety and tolerability of avalglucosidase alfa. In both trials, key secondary endpoints included motor function tests and other qualitative measures of improvement. Changes in biomarkers and anti-drug antibodies were also assessed in both trials.

**Results:**

Results for Japanese patients were representative of those from the overall populations in the COMET and Mini-COMET trials. We detail results for one Japanese patient with LOPD enrolled in the COMET trial and two Japanese patients with IOPD enrolled in the Mini-COMET trial. Importantly, avalglucosidase alfa was well tolerated at doses of both 20 mg/kg and 40 mg/kg in Japanese patients with LOPD and IOPD, respectively.

**Conclusions:**

Although the number of patients was small, avalglucosidase alfa provides an efficacy and safety profile in Japanese patients representative of the overall populations from key global clinical trials.

## 1 Introduction

Pompe disease (PD) is a rare, autosomal recessive disorder of glycogen metabolism caused by deficiency of acid-α-glucosidase (GAA), which leads to accumulation of glycogen in lysosomes [1,2]. This accumulation occurs primarily in cardiac, skeletal, and smooth muscle. PD presents on a clinical spectrum based on the age of onset range. Infantile-onset PD (IOPD) is typically rapidly progressive and severe [1,2] and typically involves cardiac manifestations (especially cardiomegaly), pulmonary manifestations, hypotonia and other motor complications [1]. If left untreated, most infants with IOPD die of cardiac or respiratory failure within one year of birth [3]. Late-onset PD (LOPD), on the other hand, can vary significantly in terms of age of onset, severity, and clinical course [4]. LOPD typically involves pulmonary manifestations, prominent skeletal symptoms such as limb-girdle weakness, and cardiomegaly is less common than in IOPD [4]. Genotype-phenotype correlation exists to some extent in IOPD and affects prognosis, including in relation to cross-reactive immunologic material (CRIM) status, which can be predicted from genotypes. CRIM-negative patients have *GAA* variants that abolishes native GAA protein synthesis or prematurely truncates the protein product whereas CRIM-positive patients are able to produce GAA protein to some recognizable degree. Immunomodulation therapy has been shown to be somewhat effective for altering the decline among CRIM-negative IOPD patients and also appears to show efficacy in CRIM-positive patients who develop high titer IgG antibodies [5,6].

Enzyme replacement therapy (ERT) with recombinant GAA is the current mainstay of treatment for both IOPD and LOPD [2,4,7]. Availability of ERT has extended survival in patients with IOPD and also improved productivity and quality of life for patients with LOPD [8,9]. ERT for patients with PD was previously limited to intravenous alglucosidase alfa (Myozyme®) 20 mg/kg every two weeks according to the approved label [10]. The long-term benefits of alglucosidase alfa established it as the current standard-of-care for patients with PD. However, progressive decline in muscle and respiratory function observed in patients receiving long-term alglucosidase alfa has been partially attributed to suboptimal uptake of ERT in skeletal muscle [11,12]. To potentially maximize the benefit of ERT for patients with PD, avalglucosidase alfa (Nexviazyme®) was developed as a modified form of alglucosidase alfa. Avalglucosidase alfa is designed for enhanced targeting of cation-independent mannose-6-phosphate (M6P) receptor-mediated uptake, the essential pathway for cellular uptake and lysosomal trafficking [13,14]. By the chemical conjugation of an oligosaccharide harbouring bis-M6P residues onto recombinant human GAA, avalglucosidase alfa has an approximately 15-times increase in levels of M6P compared with alglucosidase alfa [15,16]. Avalglucosidase alfa was approved by the US Food and Drug Administration in 2021 for patients with LOPD (one year or older) [17]. In Japan, it was approved in September 2021 for patients with LOPD (20 mg/kg every two weeks) and patients with IOPD (40 mg/kg every two weeks) [18]. Higher alglucosidase alfa doses of up to 40 mg/kg weekly have been shown to improve various measures in IOPD and LOPD without safety or immunological concerns and may be useful in patients with clinical and functional decline [19].

The efficacy and safety of avalglucosidase alfa have been demonstrated in several key studies. COMET is a Phase 3 pivotal trial in 100 patients with treatment naïve LOPD randomly assigned to avalglucosidase alfa 20 mg/kg every 2 weeks (*n* = 51) or alglucosidase alfa 20 mg/kg every 2 weeks (*n* = 49) [20]. In terms of the primary endpoint of improvement in upright forced vital capacity (FVC %) predicted, avalglucosidase alfa was non-inferior to alglucosidase alfa (difference 2.43 % [95 % CI –0.13 to 4.99]; *P* = 0.0074). Further, improvement was also noticed in the key secondary endpoint of the 6-min walking test (6MWT, difference 30.01 m [95 % CI 1.33 to 58.69]; *P* = 0.0405). Regarding IOPD, a Phase 2 trial (Mini-COMET) enrolled patients (*n* = 22) aged 1–12 years who had previously received a stable dose of alglucosidase alfa for ≥6 months and demonstrated clinical decline or sub-optimal response [21].

As reported elsewhere, Japanese patients with PD predominately possess several pathogenic variants in the *GAA* gene, including the NM_000152.5:c.546G > T (p.Thr182=) (ClinVar ID VCV000370637) pathogenic variant [22,23] as well as NM_000152.5:c.2015G > A (p.Arg672Gln) (ClinVar ID VCV000371126) and NM_000152.5:c.1798C > T (p.Arg600Cys) (ClinVar ID VCV000640911). These differ from the most frequently seen *GAA* gene variant in PD patients from Western and other Asian countries, such as NM_000152.5:c.-32-13 T > G (ClinVar ID VCV000004027), NM_000152.5:c.2238G > C (p.Trp746Cys) (ClinVar ID VCV000265160), and NM_000152.5:c.1316 T > A (p.Met439Lys) (ClinVar ID VCV000371305) noted commonly among Caucasian, Chinese, and Korean patients, respectively [[24], [25], [26], [27]]. Further, post-marketing surveillance data shows that the age at onset of LOPD may differ between patients from Japan than those in Europe and North America [28]. A known genetic difference, combined with probable region-specific lifestyle differences, suggests the need to separately verify the efficacy and safety of avalglucosidase alfa in Japanese patients enrolled in clinical trials.

Based on this background, this case series seeks to provide a detailed synopsis of Japanese patients enrolled in these pivotal trials of avalglucosidase alfa and describe efficacy and safety characteristics on the background of the overall study populations.

## 2 Methods

### 2.1 Trial designs and endpoints

This study provides detailed results concerning three Japanese patients enrolled in the COMET (for LOPD) and Mini-COMET (for IOPD) trials. Details of the design and endpoints used in the COMET and Mini-COMET trials relevant to the Japanese patients included in this case analysis have been described in full in the original publications (ClinicalTrials.gov Identifier: NCT02782741, NCT03019406) [20,21]. In brief, COMET is a Phase 3, multicenter, multinational, randomized, double blinded trial comparing efficacy and safety of intravenous avalglucosidase alfa and alglucosidase alfa in patients with treatment-naïve LOPD. The primary endpoint of the COMET trial is the change from baseline to week 49 (primary analysis period, PAP) in upright FVC % predicted. Secondary endpoints of the COMET trial of interest to the current analysis include the change from baseline to week 49 in: (i) 6MWT distance walked, (ii) quick motor function test (QMFT) total scores, (iii) 12-item short-form health survey (SF-12) physical component summary (PCS) and mental component summary (MCS) scores, (iv) upright % predicted maximal inspiratory pressure (MIP) and maximal expiratory pressure (MEP), (v) hand-held dynamometry (HHD) (lower extremity muscle strength), and (vi) safety. Mini-COMET is a Phase 2, multi-stage, randomized, open-label, multicenter, multinational, ascending-dose cohort trial primarily evaluating the safety of avalglucosidase alfa in patients with IOPD who previously treated with a stable dose of alglucosidase alfa. Patients who showed clinical decline with alglucosidase alfa treatment classified as stage 1 (Cohort 1 and 2), and patients who showed suboptimal clinical response with alglucosidase alfa treatment classified as stage 2 (Cohort 3) [21]. The primary endpoints are the number of patients with treatment-emergent adverse events (TEAEs), serious TEAEs, and adverse event of special interest from baseline to week 25 (PAP in Mini-COMET trial). Secondary endpoints of interest to the current analysis include the change from baseline to week 25 in: (i) gross motor function measure-88 (GMFM-88) total percent score, and (ii) QMFT total score, (iii) Pompe-pediatric evaluation of disability inventory (PEDI) functional skills scale: mobility domain, (iv) echocardiography (left ventricular mass [LVM] *Z*-score), and (v) eyelid position. We also report results of 6MWT and respiratory function as tertiary endpoints. Finally, data on biomarkers (creatine kinase [CK] and urinary hexose tetrasaccharide [Hex4]) and anti-drug antibodies (ADA) in the COMET and Mini-COMET trials are also reported.

For both COMET and Mini-COMET, the study was conducted in accordance with the Declaration of Helsinki and the International Council for Harmonisation guidelines for Good Clinical Practice. The study protocol was reviewed and approved by relevant institutional review boards or independent ethics committees before trial commencement. Written informed consent was obtained from the patient and/or the parent(s) or legally acceptable representatives before any study-related procedures.

### 2.2 Treatment

Patients in the COMET trial received avalglucosidase alfa (or alglucosidase alfa) 20 mg/kg every two weeks via intravenous (IV) administration. Accordingly, the Japanese patient (Patient 1) in this analysis received avalglucosidase alfa 20 mg/kg every two weeks via IV administration. Patients in the Mini-COMET trial received avalglucosidase alfa 20 mg/kg every two weeks (Cohort 1) or 40 mg/kg every two weeks (Cohort 2) via IV administration. Cohort 3 participants were randomized 1:1 to receive avalglucosidase alfa 40 mg/kg every two weeks or continue their current stable alglucosidase alfa dose. Accordingly, the Japanese patients in this analysis received avalglucosidase alfa 20 mg/kg every two weeks (Patient 2, Cohort 1) or 40 mg/kg every two weeks (Patient 3, Cohort 2) via IV administration.

## 3 Results

### 3.1 Patient baseline characteristics

In total, three Japanese patients were enrolled in the COMET (*n* = 1) and Mini-COMET (Cohort 1, n = 1; Cohort 2, n = 1) trials. The baseline characteristics of these three Japanese patients along with corresponding data for the overall treatment arms from the COMET and Mini-COMET trials are shown in Table 1 and Table 2, respectively.

**Table 1:** Baseline characteristics of a Japanese patient enrolled in the COMET trial with aggregate data for the overall population.

	COMET		
	Patient 1 (Japanese) (n = 1)	Avalglucosidase alfa-arm (n = 51)	Total population (*n* = 100)
Age, years / mean (SD)	60	46.0 (14.5)	48.1 (14.2)
Sex, male / female, n (%)	Male	27 (52.9)/24 (47.1)	52 (52.0)/48 (48.0)
Main genotype (%)	c.546G > T (homozygous)	c.-32-13 T > G (84.3 %)	c.-32-13 T > G (89.0 %)
GAA activity	1.95 pmol/punch/h (DBS); 2.09 nmol/mg protein/h (lymphocytes)	–	–
BMI, kg/m^2^/mean (SD)	30.4	26.4 (6.8)	26.5 (6.1)
Age at onset of first symptom of Pompe disease, years/mean (SD)	56.6	32.9 (16.6)^a^	35.3 (16.3)^c^
Time from first symptom to first infusion of study drug, years / mean (SD)	4.2	13.4 (11.0)^a^	13.0 (10.5)^c^
Upright FVC % predicted/mean (SD)	48.0	62.5 (14.4)	62.1 (13.3)
6MWT, m / mean (SD)	365	399.3 (110.9)	388.9 (113.5)
Upright MIP, % predicted/mean (SD)	28.4	51.7 (24.9)^b^	–
Upright MEP, % predicted/mean (SD)	54.2	59.2 (21.6)^b^	–
HHD (lower extremity), composite score/mean (SD)	1140	1330.5 (625.4)	1395.5 (616.2)
QMFT total score, mean (SD)	35	41.3 (10.2)	41.8 (10.3)
SF-12 (PCS)/mean (SD)	48.8	36.0 (7.8)	36.4 (8.6)
SF-12 (MCS)/mean (SD)	56.2	48.3 (10.1)	49.4 (9.5)
Urinary Hex4, mmol/mol creatinine/mean (SD) at the start of treatment	9.4	12.7 (10.1)	–
CK, IU/L/mean (SD) at the start of treatment	522.0	739.9 (577.6)	–

Abbreviations: 6MWT, 6-min walking test; BMI, body mass index; CK, creatine kinase; DBS, dried blood spot; FVC, forced vital capacity; GAA, acid-α-glucosidase; HHD, hand-held dynamometry; IU, international unit; MCS, mental component summary; MEP, maximal expiratory pressure; MIP, maximal inspiratory pressure; PCS, physical component summary; QMFT, quick motor function test; SD, standard deviation; SF-12, 12-item short-form health survey.

Note: ^a^n = 50; ^b^n = 48; ^c^n = 99; mean (SD) relate to overall populations (Patient 1 values are actual).

**Table 2:** Baseline characteristics of Japanese patients enrolled in the Mini-COMET trial with aggregate data for overall population.

	Mini-COMET (Cohort 1)		Mini-COMET (Cohort 2)	
	Patient 2 (Japanese) (*n* = 1)	Overall population (*n* = 6)	Patient 3 (Japanese) (n = 1)	Overall population (*n* = 5)
Age, years/mean (SD)	6.0	7.6 (3.4)	10.0	8.1 (4.1)
Sex, male/female, n (%)	Male	5 (83.3)/1 (1.7)	Female	3 (60.0)/2 (40.0)
Genotypes	c.1798C > T (homozygous)	c.[784G > A];[1935C > A;1726G > A], c.[1062C > G;1286 A > G];[1935C > A;1726G > A], c.[1222 A > G];[1927G > A], c.[236_246del];[655G > A], c.[1798C > T];[1798C > T], c.[1000G > T];[271G > A;307 T > G]	c.2015G > A (homozygous)	c.[1935C > A;1726G > A];[2842INST], c.[1935C > A;1726G > A];[1935C > A;1726G > A], c.[1193del];[1927G > A], c.[2015G > A];[2015G > A], c.[2646 + 2 T > A]
GAA activity	2.79 pmol/punch/h (DBS)		5.02 nmol/mg protein/h (lymphocyte); 2.82 nmol/mg protein/h (skin fibroblast)	
Height, cm/mean (SD)	114	123.4 (22.9)	131	126.6 (30.5)
Weight, kg/mean (SD)	17	28.5 (15.0)	21	32.5 (21.5)
BMI, kg/m^2^/mean (SD)	13.2	17.5 (4.2)	12.0	18.3 (5.6)
CRIM negative, yes/n (%)	0	1 (17)	0	1 (20)
Age at onset of first symptom of Pompe disease, months/mean (SD)	0.0^a^	1.2 (1.7)	6.5	3.3 (2.9)
Age at diagnosis of Pompe disease, months/mean (SD)	1.6	1.9 (2.1)	7.2	4.3 (3.8)
Age at first treatment with alglucosidase alfa, months/mean (SD)	1.7	2.9 (2.4)	10.4	5.3 (4.8)
Time from first symptom to first treatment with alglucosidase alfa, months/mean (SD)	1.7	1.7 (1.4)	3.9	2.0 (2.2)
Cardiomegaly, yes / n (%)	1	6 (100)	1	5 (100)
Congestive heart failure, yes/n (%)	0	0 (0)	1	1 (20.0)
GMFM-88, total percent score/mean (SD)	57.6	54.8 (31.4)	96.3	67.4 (33.8)
QMFT total score/mean (SD)	19	23.3 (14.8)	52	31.2 (20.0)
Urinary Hex4, mmol/mol creatinine/mean (SD) at start of treatment	105.9	80.3 (48.4)	78.9	63.4 (30.7)
CK, IU/L/mean (SD) at start of treatment	2607.0	1102.2 (932.4)	1477.0	1444.8 (164.2)

Abbreviations: BMI, body mass index; CK, creatine kinase; CRIM, cross-reactive immunologic material; DBS, dried blood spot; GAA, acid-α-glucosidase; GMFM-88, gross motor function measure-88; Hex4, hexose tetrasaccharide; HHD, hand-held dynamometry; IU, international unit; QMFT, quick motor function test; SD, standard deviation.

Expanded variant nomenclature: NM_000152.5:c.784G > A (p.Glu262Lys) (ClinVar ID VCV000188806); NM_000152.5:c.1062C > T (p.Tyr354=) (ClinVar ID VCV001548389); NM_000152.5:c.1286A > G (p.Gln429Arg) (ClinVar ID VCV000284497); NM_000152.5:c.1222A > G (p.Met408Val) (ClinVar ID VCV000371235); NM_000152.5:c.236_246del (p.Pro79fs) (ClinVar ID VCV000371302); NM_000152.5:c.1000G > T (p.Gly334Cys) (ClinVar ID VCV000284864); NM_000152.5:c.1935C > A (p.Asp645Glu) (ClinVar ID VCV000004029); NM_000152.5:c.1726G > A (p.Gly576Ser) (ClinVar ID VCV000092467); NM_000152.5:c.1193del (p.Leu398fs) (ClinVar ID VCV000371457); NM_000152.5:c.2646 + 2 T > A (ClinVar ID VCV000188924); NM_000152.5:c.1927G > A (p.Gly643Arg) (ClinVar ID VCV000004023); NM_000152.5:c.655G > A (p.Gly219Arg) (ClinVar ID VCV000189065); NM_000152.5:c.271G > A (p.Asp91Asn) (ClinVar ID VCV000004020); NM_000152.5:c.307T > G (p.Cys103Gly) (ClinVar ID VCV000092483)

^a^ Elevated serum CK and hypertrophic cardiomyopathy on echocardiography; mean (SD) and n (%) relate to the overall populations (Patient 2 and 3 values are actual).

Patients in Mini-COMET were treated with avalglucosidase alfa 20 mg/kg every two weeks (Cohort 1) or 40 mg/kg every two weeks (Cohort 2 and Cohort 3 avalglucosidase alfa arm). Results from this trial found that, despite TEAEs being noted in all enrolled patients, avalglucosidase alfa was well-tolerated, and no additional safety concerns were noted in patients switching from alglucosidase alfa to avalglucosidase alfa. In terms of efficacy, avalglucosidase alfa led to a stable or improved response in terms of cardiac or motor function. GMFM-88 total percent score improved modestly in all cohorts (Cohort 1: median at baseline, 56.25; median change from baseline to week 25, 1.68; Cohort 2: median at baseline, 86.63; median change from baseline to week 25, 0.83; Cohort 3: median at baseline, 59.63, median change from baseline to week 25, 7.68), with high interindividual variability. QMFT total score improved in Cohorts 2 (median at baseline, 38.00; median change from baseline to week 25, 2.00) and 3 (median at baseline, 24.00; median change from baseline to week 25, 4.00), whereas mean score in Cohort 1 remained stable (median at baseline, 25.00; median change from baseline to week 25, −0.50). No decline from baseline was observed in LVM *Z*-score during the primary analysis period, while a sole participant with abnormal LVM Z-score (> 2) at baseline improved to normal range.

Baseline characteristics of Patient 1, a Japanese adult enrolled in the COMET trial, were generally within the standard deviation (SD) spread of the overall population. Similarly, baseline characteristics of Patient 2 and Patient 3 from the Mini-COMET trial were relatively typical in relation to the overall trial population, suggesting these two patients with IOPD were representative cases for comparison at baseline.

### 3.2 Patient 1 (COMET trial)

Patient 1 was a 60-year-old male with LOPD with a baseline body weight of 84 kg (body mass index [BMI] 30.4 kg/m^2^) (Table 1). Patient 1 presented initially with a chief complaint of lower limb muscle weakness and elevated CK levels. Prior to this, the patient had a history of muscle weakness in the lower back and lower limbs, which gradually led to difficulty standing up as well as shortness of breath and discomfort sleeping on their side. One year before diagnosis, the patient had elevated CK levels and trunk/proximal muscle weakness were noted. Statins were discontinued due to suspected influence on CK levels although detailed blood test data revealed that CK levels were elevated even before starting statin therapy. Instead, based on the gradual progression of muscle weakness in the lumbar girdle, myopathy was suspected. Echocardiogram found no abnormalities in left ventricular wall motion and an ejection fraction of 69 %. Electrocardiogram (ECG) showed that the heart rate was regular at 76 bpm, and no abnormalities were detected. Electromyogram found rapid recruitment in all tested muscles with fibrillation potential/positive sharp waves (1+) in the deltoid, biceps brachii, and quadriceps muscles at rest. The diagnosis was provisionally suspected by reduction in GAA enzymatic activity in dried blood spot (DBS, performed as screening) of 1.95 pmol/punch/h and more definitively confirmed by genetic testing and reduction in GAA enzymatic activity in lymphocytes (2.085 nmol/mg protein/h). However, there was no relevant family history, evidence of speech difficulties, gastroesophageal reflux, sleep apnea, or scoliosis and hearing loss was not recorded at diagnosis. Based on genetic testing, the homozygous NM_000152.5:c.546G > T pathogenic variant of the *GAA* gene was detected in Patient 1. NM_000152.5:c.546G > T has previously been reported as the most common pathogenic variant in Japan [22]. This pathogenic variant resulted in a nucleotide change but, being a leaky splice mutation, it resulted in a low-level expression of GAA [29]. Age at onset of first symptoms was 56.6 years and treatment exposure duration of avalglucosidase alfa during the PAP was 11.5 months (49 weeks) with treatment continued additional 7.3 months (extended treatment period). Patient 1 was treatment-naïve at the start of randomly assigned avalglucosidase alfa treatment.

**Fig. 1:**
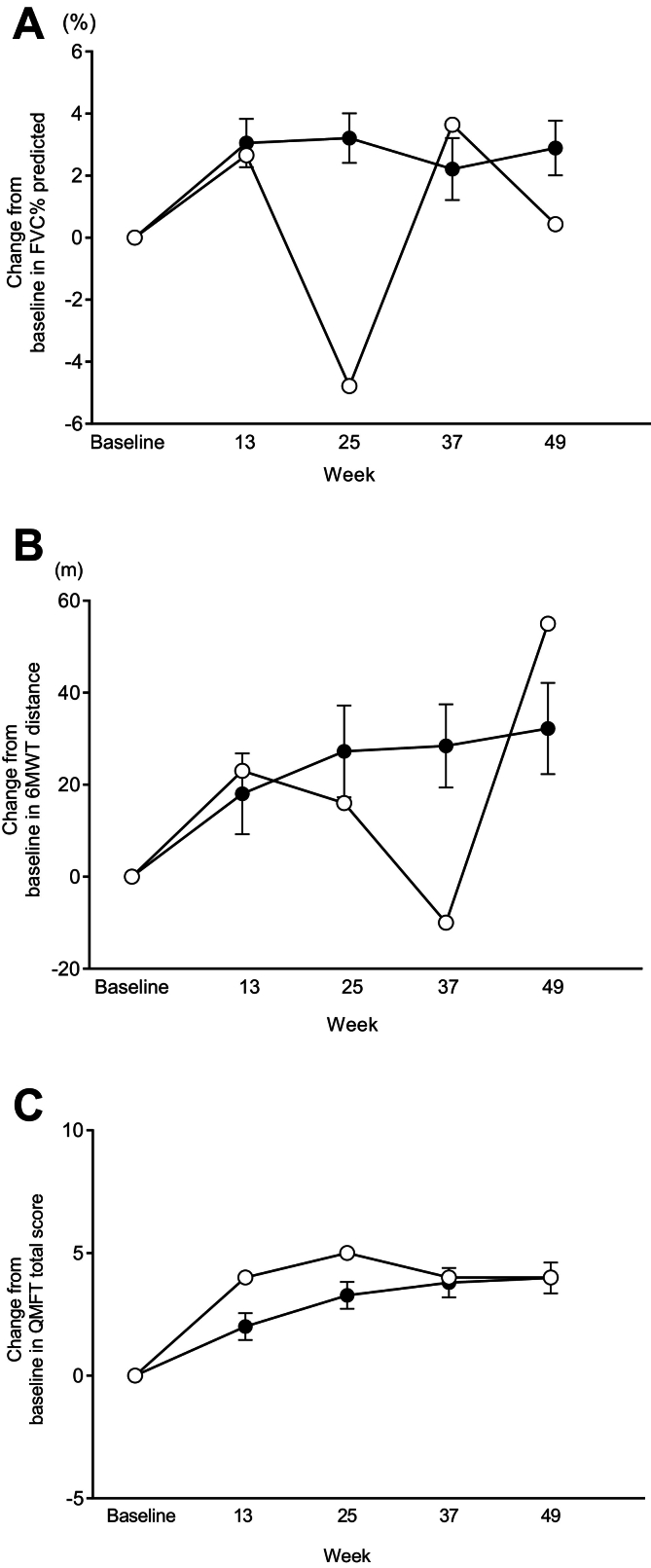
Changes from baseline in (A) FVC % predicted, (B) 6MWT distance, and (C) QMFT total score in Patient 1 (○) and the mean (SE) changes in the avalglucosidase alfa-arm (•) from the COMET trial.

Primary endpoint data for Patient 1 showed that changes in FVC % predicted were different to those of the overall population at evaluation time points with a change from baseline to week 49 of 0.43 %. In contrast, the overall population showed a gradual improvement over this timeframe (Fig. 1A). Changes in 6MWT also showed a decline followed by improvement at week 49, which eclipsed the gradual improvement seen in the overall population (Fig. 1B). The actual change in 6MWT and 6MWT % predicted from baseline to week 49 were 55.0 m and 8.26 %, respectively. The change from baseline to week 49 in QMFT total score in this Japanese patient (4.0) was similar to mean values of the overall population, which showed an improvement of approximately 5 points (Fig. 1C).

The change in MIP % and MEP % predicted values from baseline to week 49 were 0.12 % and 15.01 %, respectively. HHD values improved initially from baseline but declined later in treatment (change in HHD [lower extremity] composite score from baseline to week 49 was −91.0). Both the SF-12 PCS and SF-12 MCS score were improved at week 49 compared with baseline (change in SF-12 PCS from baseline, 4.68; change in SF-12 MCS from baseline, 0.70). Changes in efficacy parameters for Patient 1 are summarized in Supplementary Table 1.

**Fig. 2:**
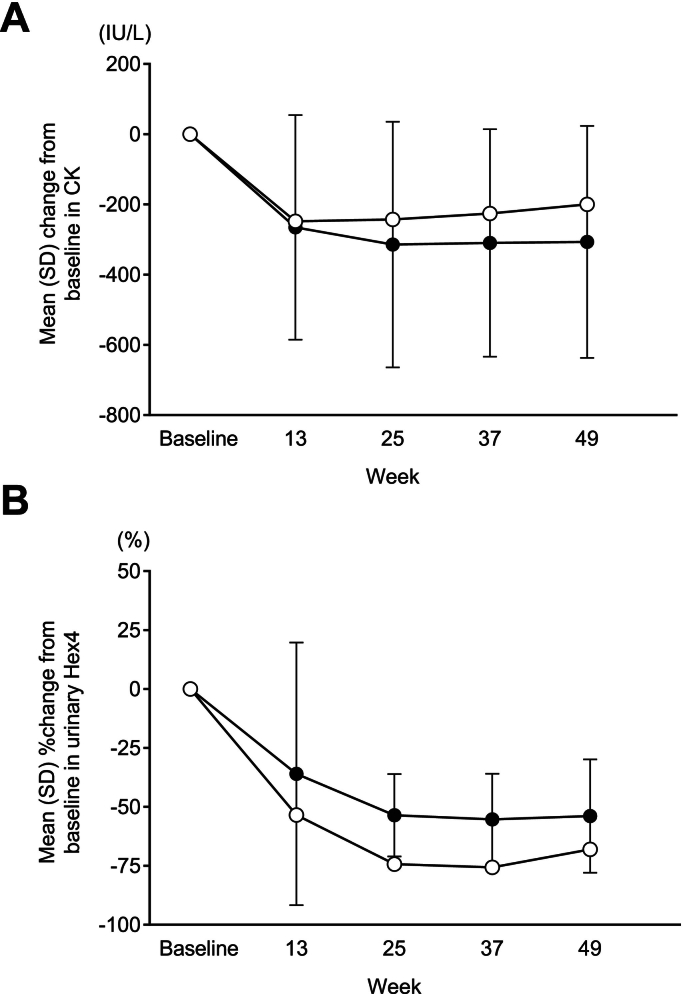
Change in (A) CK and (B) urinary Hex4 in Patient 1 (○) and the mean (SD) changes in the avalglucosidase alfa-arm (•) from the COMET trial.

Changes in biomarker, including reductions in CK and urinary Hex4, were also similar to the mean values for the overall population (Fig. 2).

Motor functions were improved after starting avalglucosidase alfa treatment. After 4 days, the patient felt lighter on his feet and, at 10 weeks, he felt it was easier to stand up. At 16 weeks, the patient felt it was easier to stand up, walk, and adopt a spine position for 1–2 min, which were completely impossible previously. Further, although the patient struggled to walk 300 m and felt short of breath before starting the clinical trial, he was able to walk 1 km without being out of breath at week 22 and became able to pick things up from the floor at week 36.

Overall, avalglucosidase alfa 20 mg/kg was well tolerated in this patient. There were no TEAEs leading to treatment discontinuation, infusion-associated reactions, treatment-emergent anaphylactic reactions/hypersensitivity or other immune-mediated reactions in this patient. TEAEs reported in this patient were nasopharyngitis, skin papilloma, dyslipidaemia, hyperuricaemia, dental caries, and pain in extremity, which were all mild and considered as not related to avalglucosidase alfa treatment. This patient developed treatment-emergent ADA, with an intermediate peak titer (3200, Supplementary Fig. 1), and also developed neutralizing antibodies between week 13 and 25 that inhibited avalglucosidase alfa catalytic activity. These neutralizing antibodies became negative between week 29 and 49.

### 3.3 Patient 2 (Mini-COMET, cohort 1)

Patient 2 from Cohort 1 of the Mini-COMET trial was a 6-year-old male with baseline body weight of 17 kg (BMI 13.2 kg/m^2^) (Table 2). Genetic analysis revealed that Patient 2 had the homozygous NM_000152.5:c.1798C > T pathogenic variant, which is a common variant in Japanese patients and known to cause IOPD. This variant can produce the GAA protein with almost no activity [30], indicating that patients who have this variant are CRIM-positive. Patient 2 had an older biological sister with IOPD and, although no neurological symptoms were observed and muscle tone was normal at birth, hyperCKemia, and myocardial hypertrophy via echocardiography were detected at 0 months. The diagnosis of PD was confirmed at 1.6 months. Alglucosidase alfa treatment was started immediately after diagnosis at 1.6 months. Immunomodulation was not conducted in this patient. At 6 years of age, this patient demonstrated clinical decline with alglucosidase alfa, enrolled in the Mini-COMET trial and started avalglucosidase alfa. This patient presented clinically with the following conditions or symptoms: cardiomegaly but no evidence of congestive heart failure (CHF); an enlarged tongue; hearing loss; muscle weakness present in both the upper and lower extremities as well as joint contractures; need for assistance devices (including a wheelchair and foot orthoses) for ambulation; initial need for non-invasive nocturnal ventilator assistance for respiratory failure at 6 years, 3 months (5 months before starting avalglucosidase alfa). Mild motor and speech developmental delays were also observed. The child started walking and speaking at 1 year and 7 months. CK and Hex4 levels were not measured at birth. However, the CK level was 868 U/L at 27 days old, whereas Hex4 was not measured at this point. ECG at 44 days old showed left ventricular hypertrophy. Hearing impairment was noted during episodes of worsening otitis media since the age of 5 years. However, the condition improved with treatment for otitis media and no hearing aid was required. Avalglucosidase alfa continued for a total of 94 weeks, including the 26 weeks of the PAP.

Regarding key safety outcome data, avalglucosidase alfa 20 mg/kg was well tolerated in this patient with no severe TEAEs and no inducement of ADA (Table 3). Patient 2 reported the following TEAEs: dental caries, myalgia, presyncope, lymph node pain, and alopecia areata, which were all considered mild and not related to avalglucosidase alfa treatment.

**Table 3:** Summary of adverse events for Japanese patients enrolled in the COMET and Mini-COMET trials.

		COMET		Mini-COMET			
				Cohort 1 (20 mg/kg)		Cohort 2 (40 mg/kg)	
		Patient 1 (Japanese) (n = 1)	Avalglucosidase alfa-arm (*n* = 51)	Patient 2 (Japanese) (n = 1)	Overall Population (*n* = 6)	Patient 3 (Japanese) (n = 1)	Overall Population (n = 5)
TEAEs, n (%)		1	44 (86.3)	1	5 (83.3)	1	5 (100)
TEAEs potentially related to treatment, n (%)		0	23 (45.1)	0	0	0	2 (40.0)
Severe TEAEs, n (%)		0	8 (15.7)	0	1 (16.7)	0	3 (60.0)
Severe TEAEs potentially related to treatment, n (%)		0	1 (2.0)	0	0	0	0
TEAEs leading to study withdrawal		0	0	0	0	0	0
TEAEs leading to study death, n (%)		0	0	0	0	0	0
Infusion associated reactions, n (%)		0	13 (25.5)	0	0	0	2 (40.0)
COMET	ADA status, n (%)						
	Always negative	0	2 (3.9)				
	Ever positive with negative baseline	1	47 (92.2)	–	–	–	–
	Positive at baseline	0	2 (3.9)				
	ADA peak titer, n (%)						
	Negative	0	2 (3.9)				
	100–800	0	17 (33.3)	–	–	–	–
	1,600–6,400	1	20 (39.2)				
	≥ 12,800	0	10 (19.6)				
Mini-COMET	Treatment-induced ADA positive, n (%)	–	–	0	0	0	1 (20.0)^a^

Abbreviations: ADA, anti-drug antibodies; TEAEs, treatment-emergent adverse events.

^a^ Peak titer = 800.

Regarding efficacy, changes from baseline to week 25 in GMFM-88 total percent score and QMFT total score were similar to mean values for the overall population (Supplementary Table 2 and Supplementary Fig. 2A).

**Fig. 3:**
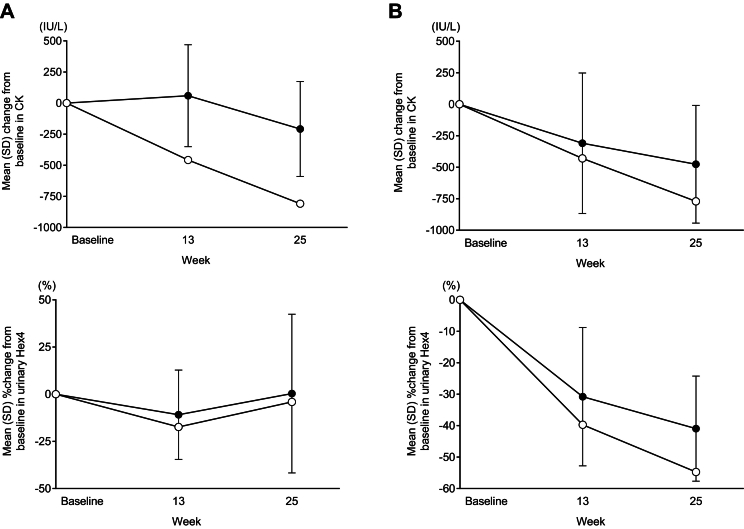
Change in CK and urinary Hex4 in (A) Patient 2 and (B) Patient 3 (○) together with mean (SD) changes in the overall population in each cohort (•) from the Mini-COMET trial.

The change from baseline to week 25 in GMFM-88 total percent score and QMFT total score were 2.96 % and −2, respectively. There was little change from baseline to week 25 in the Pompe-PEDI score in this Japanese patient (0.3). Echocardiography results found that there was an improvement in LVM *Z*-score during the PAP with the LVM Z-score at baseline (−1.4) increasing to −0.8 at week 25 (Supplementary Table 2). Regarding eyelid position, this patient had ptosis in both eyes at baseline and week 25. Pulmonary function tests were not required for this patient given he is using ventilatory assistance while sleeping according to the clinical trial protocol. Similarly, 6MWT data at week 25 was not obtained in this patient as he is using assistance devices for ambulation. Reductions in CK from baseline to week 25 (2607 IU/L to 1798 IU/L) exceeded those seen in the overall trial population (mean 1102.2 IU/L to 893.5 IU/L) whereas changes in urinary Hex4 closely resembled those of the overall trial population (Fig. 3A).

### 3.4 Patient 3 (Mini-COMET, cohort 2)

Patient 3 from Cohort 2 of the Mini-COMET trial was a 10-year-old female (BMI 12.0 kg/m^2^) (Table 2). Genetic analysis revealed that Patient 3 had the homozygous NM_000152.5:c.2015G > A pathogenic variant, which is known to cause IOPD. This variant can produce the GAA protein with almost no enzymatic activity [30], indicating that patients who have this variant are CRIM-positive. Age at onset of first symptoms was 6.5 months and PD was diagnosed at 7.2 months, with alglucosidase alfa treatment started at 10.4 months. The presenting symptoms at 6.5 months were delayed neck control and poor body weight gain as a result of poor feeding. At onset, Patient 3 also had cardiomegaly with evidence of CHF (Class II), hepatomegaly, and an enlarged tongue but had no hearing loss. Other symptoms included tachycardia and tachypnea. At diagnosis, CK was 449 IU/L whereas Hex4 was not measured. ECG confirmed the presence of sinus tachycardia (150 bpm) and showed other features, including shortened PQ interval, high QRS voltage in all leads, upright positive T wave in V1 suggestive of right ventricular hypertrophy, and abnormal Q waves in several leads. Age of first speech and walking was 18 months, suggesting the catch-up of developmental motor delay and speech delay. Immunomodulation was not conducted in this patient. At 10 years of age, this patient demonstrated clinical decline with alglucosidase alfa, enrolled in the Mini-COMET trial and started avalglucosidase alfa. Clinical decline was observed in relation to the following conditions or symptoms: muscle weakness (mainly manifested as a delay in holding up the head), cardiomegaly with evidence of CHF (Class II), hepatomegaly, and an enlarged tongue but had no hearing loss. The duration of PAP administration was 6 months. Unlike Patient 2 described above, muscle weakness was mild (difficulty holding up head in supine position, slowing in running ability), and the patient was ambulatory without assistance. The anti-alglucosidase alfa IgG antibody titers increased from 200 at 28 months to 400 at 34 months, peaked at 1,600 at 47 months, then decreased to 400 at 51 months and to 200 at 61 months, remaining below 200 thereafter. Detailed data regarding neutralizing antibody titers are not available.

Avalglucosidase alfa 40 mg/kg was well tolerated with no severe TEAEs and no inducement of ADA (Table 3). Patient 3 reported the following TEAEs: skin abrasion, ligament sprain, arthropod sting, sunburn, arthralgia, musculoskeletal pain, erythema, infusion site swelling, abdominal pain, thermal burn, contusion, oropharyngeal pain, vomiting, back pain, headache, foot deformity, and epistaxis. All TEAEs were considered mild and not related to avalglucosidase alfa treatment.

Regarding efficacy, changes from baseline to week 25 in GMFM-88 total percent score and QMFT total score were similar to mean values for the overall population (Supplementary Table 2 and Supplementary Fig. 2B).

Specifically, the change from baseline to week 25 in GMFM-88 total percent score and QMFT total score were 0.83 % and 2, respectively. Pompe-PEDI scores were 0 at baseline and week 25 with no change over this period. There was little change in the echocardiography parameters (LVM *Z*-score at baseline and week 25 were −0.6 and −0.7, respectively) (Supplementary Table 2). No ptosis was seen in either eye at baseline and week 25. Regarding respiratory function, FVC % predicted, MIP % predicted, and MEP % predicted changed from baseline values of 60 %, 9.65 %, and 18.95 %, respectively, to week 25 values of 64 %, 93.67 %, and 74.73 %, respectively (equating to changes from baseline to week 25 of 4 %, 84.02 %, and 55.78 %, respectively). There was also an improvement in 6MWT results, from 452 m at baseline to 511 m at week 25. Changes in CK and urinary Hex4 were also similar to mean values for the overall population (Fig. 3B).

## 4 Discussion

Individual results of this case series suggest that responses to avalglucosidase alfa in Japanese patients with LOPD and IOPD are representative of those seen in overall trial populations. For example, although the changes in FVC % predicted for Patient 1 fluctuated and showed minimal change from baseline at Week 49, changes in 6MWT and QMFT total score were also representative of those for the overall population. Similarly, results for GMFM-88 total percent score and QMFT total score observed in Patient 2 and Patient 3 were similar to those for the overall population. However, as in all clinical trials, individual patient results are not necessarily representative of results from the overall trial population and so these results also need to be considered in that light. Indeed, one of the significant findings of these detailed analyses of individual patients is the fact that three types of *GAA* gene pathogenic variants observed in Japanese cases have not been identified in other cases. However, where there are similarities between individual and overall patient data, we believe it is reasonable to highlight these.

In terms of safety, avalglucosidase alfa was well tolerated in all Japanese patients (Table 3). In particular, the Japanese patients enrolled in the Mini-COMET trial who received avalglucosidase alfa 40 mg/kg also had an acceptable tolerability profile. These results are similar to those of the COMET and Mini-COMET trials. In the COMET trial, TEAEs were common, occurring in 86.3 % of patients, but the incidence of severe TEAEs potentially related to treatment was low (*n* = 1, 2.0 %). Presence of ADA was also common with 92.2 % of patients experiencing inducement of ADA after initially being negative at baseline. In the Mini-COMET trial, 83.3 % and 100.0 % of patients experienced TEAEs in Cohort 1 and Cohort 2, respectively. However, ADA positivity was low (only one patient in Cohort 2), which concurs with the experience of Japanese patients with IOPD in this series of cases.

Regarding efficacy, individual results in the Japanese patients included in the COMET and Mini-COMET trials resembled mean values for the overall populations. This is despite the presence of individual variation in the presence or absence of improvement in certain parameters and the quantitative extent of changes. The Japanese patient enrolled in the COMET trial showed a similar improvement in respiratory function at the final endpoint of the PAP although the course of this involved an unexplained deterioration at week 25. Changes in the 6MWT also reflected this pattern and suggest a temporary worsening in the condition of the patient during the course of the study. Changes in efficacy parameters similar to mean values for the overall population included QMFT total score, and SF-12 PCS score. In terms of biomarkers, changes in CK and urinary Hex4 levels in this Japanese patient were similar to those seen in the overall population. Improvements in the Japanese patients enrolled in the Mini-COMET trial varied. In both Japanese patients from the Mini-COMET trial, efficacy improvements similar to mean values for the overall population was noted for QMFT. Notably, improvements in biomarkers were noted in the patient who received avalglucosidase alfa 40 mg/kg. Neither patient showed changes in eyelid position although Patient 3 demonstrated a slight improvement in respiratory function and walking distance by the end of the PAP.

The limitations of this small case series are relatively obvious in that the small number of Japanese patients included does not allow firm conclusions to be drawn in this population. However, to the extent in which changes resembled those of the overall population, these results provide a strong suggestion that clinicians may expect a similar effect from avalglucosidase alfa as noted in the primary trials. Further validation of this suggestion is required in properly powered studies.

We conclude that avalglucosidase alfa provides an efficacy and safety profile in Japanese patients representative of that of the overall populations from key international trials. Importantly, no new or unique safety signals were detected among Japanese patients with LOPD or IOPD.

## Funding

This study was funded by Sanofi K.K.

## CRediT authorship contribution statement

**Madoka Mori-Yoshimura:** Writing – review & editing, Supervision, Investigation. **Hirotaka Ohki:** Writing – review & editing, Supervision, Investigation. **Hideaki Mashimo:** Writing – review & editing, Supervision, Investigation. **Kenji Inoue:** Writing – review & editing, Supervision, Investigation. **Satoko Kumada:** Writing – review & editing, Supervision, Investigation. **Takashi Kiyono:** Writing – review & editing, Conceptualization. **Akihiro Arimori:** Writing – review & editing, Conceptualization. **Mitsunobu Ikeda:** Writing – review & editing, Conceptualization. **Hirofumi Komaki:** Writing – review & editing, Supervision, Investigation, Conceptualization.

## Declaration of competing interest

H.K. has received grants or contracts as well as payment or honoraria for lectures, presentations, speakers' bureaus, manuscript writing or educational events from Sanofi K.K. T.K., A.A. M.I. are employees of Sanofi K.K. All other authors report no conflicts of interest.

## Data Availability

The data concerns individual patients and cannot be shared for confidentiality reasons.

## Appendix A Supplementary data

Supplementary material Image 1

## References

[1] Dasouki M., Jawdat O., Almadhoun O., Pasnoor M., McVey A.L., Abuzinadah A., Herbelin L., Barohn R.J., Dimachkie M.M. (2014). Pompe disease: literature review and case series. Neurol. Clin..

[2] Kishnani P.S., Steiner R.D., Bali D., Berger K., Byrne B.J., Case L.E., Crowley J.F., Downs S., Howell R.R., Kravitz R.M., Mackey J., Marsden D., Martins A.M., Millington D.S., Nicolino M., O’grady G., Patterson M.C., Rapoport D.M., Slonim A., Spencer C.T., Tifft C.J., Watson M.S. (2006). Pompe disease diagnosis and management guideline. Genet. Med..

[3] van den Hout H.M., Hop W., van Diggelen O.P., Smeitink J.A., Smit G.P., Poll-The B.T., Bakker H.D., Loonen M.C., de Klerk J.B., Reuser A.J., van der Ploeg A.T. (2003). The natural course of infantile Pompe's disease: 20 original cases compared with 133 cases from the literature. Pediatrics.

[4] Cupler E.J., Berger K.I., Leshner R.T., Wolfe G.I., Han J.J., Barohn R.J., Kissel J.T. (2012). Consensus treatment recommendations for late-onset Pompe disease. Muscle Nerve.

[5] Banugaria S.G., Prater S.N., Patel T.T., Dearmey S.M., Milleson C., Sheets K.B., Bali D.S., Rehder C.W., Raiman J.A., Wang R.A., Labarthe F., Charrow J., Harmatz P., Chakraborty P., Rosenberg A.S., Kishnani P.S. (2013). Algorithm for the early diagnosis and treatment of patients with cross reactive immunologic material-negative classic infantile pompe disease: a step towards improving the efficacy of ERT. PLoS One.

[6] Desai A.K., Li C., Rosenberg A.S., Kishnani P.S. (2019). Immunological challenges and approaches to immunomodulation in Pompe disease: a literature review. Ann. Transl. Med..

[7] Chien Y.H., Hwu W.L., Lee N.C. (2013). Pompe disease: early diagnosis and early treatment make a difference. Pediatr. Neonatol..

[8] Kishnani P.S., Hwu W.L., Mandel H., Nicolino M., Yong F., Corzo D. (2006). A retrospective, multinational, multicenter study on the natural history of infantile-onset Pompe disease. J. Pediatr..

[9] Güngör D., Kruijshaar M.E., Plug I., D’Agostino R.B., Hagemans M.L., van Doorn P.A., Reuser A.J., van der Ploeg A.T. (2013). Impact of enzyme replacement therapy on survival in adults with Pompe disease: results from a prospective international observational study. Orphanet J. Rare Dis..

[10] Genzyme Corporation (2024). MYOZYME® (alglucosidase alfa) Approved Prescribing Information. https://www.accessdata.fda.gov/drugsatfda_docs/label/2019/125141s222lbl.pdf.

[11] Prater S.N., Banugaria S.G., DeArmey S.M., Botha E.G., Stege E.M., Case L.E., Jones H.N., Phornphutkul C., Wang R.Y., Young S.P., Kishnani P.S. (2012). The emerging phenotype of long-term survivors with infantile Pompe disease. Genet. Med..

[12] Do H.V., Khanna R., Gotschall R. (2019). Challenges in treating Pompe disease: an industry perspective. Ann. Transl. Med..

[13] Braulke T., Bonifacino J.S. (2009). Sorting of lysosomal proteins. Biochim. Biophys. Acta.

[14] Wisselaar H.A., Kroos M.A., Hermans M.M., van Beeumen J., Reuser A.J. (1993). Structural and functional changes of lysosomal acid alpha-glucosidase during intracellular transport and maturation. J. Biol. Chem..

[15] Zhou Q., Avila L.Z., Konowicz P.A., Harrahy J., Finn P., Kim J., Reardon M.R., Kyazike J., Brunyak E., Zheng X., Patten S.M., Miller R.J., Pan C.Q. (2013). Glycan structure determinants for cation-independent mannose 6-phosphate receptor binding and cellular uptake of a recombinant protein. Bioconjug. Chem..

[16] Zhu Y., Jiang J.L., Gumlaw N.K., Zhang J., Bercury S.D., Ziegler R.J., Lee K., Kudo M., Canfield W.M., Edmunds T., Jiang C., Mattaliano R.J., Cheng S.H. (2009). Glycoengineered acid alpha-glucosidase with improved efficacy at correcting the metabolic aberrations and motor function deficits in a mouse model of Pompe disease. Mol. Therapy.

[17] Sanofi (2024). FDA approves Nexviazyme® (avalglucosidase alfa-ngpt), an Important New Treatment Option for Late-Onset Pompe Disease [Press Release]. https://www.sanofi.com/en/media-room/press-releases/2021/2021-08-06-15-42-21-2276588.

[18] Sanofi (2024). Sanofi Announces Results of CHMP Re-examination of the New Active Substance Status for Avalglucosidase Alfa, a Potential New Standard of Care for the Treatment of Pompe Disease. https://www.sanofi.com/en/media-room/press-releases/2021/2021-11-12-11-38-38-2333221.

[19] Khan A.A., Case L.E., Herbert M., DeArmey S., Jones H., Crisp K., Zimmerman K., ElMallah M.K., Young S.P., Kishnani P.S. (2020). Higher dosing of alglucosidase alfa improves outcomes in children with Pompe disease: a clinical study and review of the literature. Genet. Med..

[20] Diaz-Manera J., Kishnani P.S., Kushlaf H., Ladha S., Mozaffar T., Straub V., Toscano A., van der Ploeg A.T., Berger K.I., Clemens P.R., Chien Y.H., Day J.W., Illarioshkin S., Roberts M., Attarian S., Borges J.L., Bouhour F., Choi Y.C., Erdem-Ozdamar S., Goker-Alpan O., Kostera-Pruszczyk A., Haack K.A., Hug C., Huynh-Ba O., Johnson J., Thibault N., Zhou T., Dimachkie M.M., Schoser B. (2021). Safety and efficacy of avalglucosidase alfa versus alglucosidase alfa in patients with late-onset Pompe disease (COMET): a phase 3, randomised, multicentre trial. Lancet Neurol..

[21] Kishnani P.S., Kronn D., Brassier A., Broomfield A., Davison J., Hahn S.H., Kumada S., Labarthe F., Ohki H., Pichard S., Prakalapakorn S.G., Haack K.A., Kittner B., Meng X., Sparks S., Wilson C., Zaher A., Chien Y.-H. (2023). Safety and efficacy of avalglucosidase alfa in individuals with infantile-onset Pompe disease enrolled in the phase 2, open-label Mini-COMET study: The 6-month primary analysis report. Genet. Med..

[22] Fukuhara Y., Fuji N., Yamazaki N., Hirakiyama A., Kamioka T., Seo J.H., Mashima R., Kosuga M., Okuyama T. (2018). A molecular analysis of the GAA gene and clinical spectrum in 38 patients with Pompe disease in Japan. Mol. Genet. Metabol. Rep..

[23] Momosaki K., Kido J., Yoshida S., Sugawara K., Miyamoto T., Inoue T., Okumiya T., Matsumoto S., Endo F., Hirose S., Nakamura K. (2019). Newborn screening for Pompe disease in Japan: report and literature review of mutations in the GAA gene in Japanese and Asian patients. J. Hum. Genet..

[24] Kroos M.A., Van der Kraan M., Van Diggelen O.P., Kleijer W.J., Reuser A.J., Van den Boogaard M.J., Ausems M.G., Ploos van Amstel H.K., Poenaru L., Nicolino M. (1995). Glycogen storage disease type II: frequency of three common mutant alleles and their associated clinical phenotypes studied in 121 patients. J. Med. Genet..

[25] Laforêt P., Nicolino M., Eymard P.B., Puech J.P., Caillaud C., Poenaru L., Fardeau M. (2000). Juvenile and adult-onset acid maltase deficiency in France: genotype-phenotype correlation. Neurology.

[26] Liu X., Wang Z., Jin W., Lv H., Zhang W., Que C., Huang Y., Yuan Y. (2014). Clinical and GAA gene mutation analysis in mainland Chinese patients with late-onset Pompe disease: identifying c.2238G > C as the most common mutation. BMC Med. Genet..

[27] Park H.D., Lee D.H., Choi T.Y., Lee Y.K., Lee S.Y., Kim J.W., Ki C.S., Lee Y.W. (2013). Three patients with glycogen storage disease type II and the mutational spectrum of GAA in Korean patients. Ann. Clin. Lab. Sci..

[28] Nagura H., Hokugo J., Ueda K. (2019). Long-term observation of the safety and effectiveness of enzyme replacement therapy in Japanese patients with pompe disease: Results from the post-marketing surveillance. Neurol. Therapy.

[29] Maimaiti M., Takahashi S., Okajima K., Suzuki N., Ohinata J., Araki A., Tanaka H., Mukai T., Fujieda K. (2009). Silent exonic mutation in the acid-alpha-glycosidase gene that causes glycogen storage disease type II by affecting mRNA splicing. J. Hum. Genet..

[30] Tsujino S., Huie M., Kanazawa N., Sugie H., Goto Y., Kawai M., Nonaka I., Hirschhorn R., Sakuragawa N. (2000). Frequent mutations in Japanese patients with acid maltase deficiency. Neuromuscul. Disord..

