# Supplementary material for: Efficacy and safety of avalglucosidase alfa in Japanese patients with late-onset and infantile-onset Pompe diseases: A case series from clinical trials
Source: Mol Genet Metab Rep. 2024 Dec 27;42:101163. doi: 10.1016/j.ymgmr.2024.101163 (PMC11743810; doi:10.1016/j.ymgmr.2024.101163)

**Supplementary Material**

Madoka Mori-Yoshimura, et al. Efficacy and safety of avalglucosidase alfa in Japanese patients with late-onset and infantile-onset Pompe diseases: a case series from clinical trials. Molecular Genetics and Metabolism.

Contents

[Supplementary Table 1. Baseline / change in efficacy parameters (primary and secondary endpoints) from baseline to week 49 in a Japanese patient and overall population from the COMET trial 2](#_Toc175130953)

[Supplementary Table 2. Baseline / changes in efficacy parameters (secondary endpoints) from baseline to week 25 in Japanese patients and overall population from the Mini-COMET trial 4](#_Toc175130954)

[Supplementary Fig. 1. Course of ADA titer in Patient 1 5](#_Toc175130955)

[Supplementary Fig. 2. GMFM-88 total percent score and QMFT total score in (A) Patient 2 (B) Patient 3 (⭘) and the mean (SD) in the overall population in each cohort (⚫) from the Mini-COMET trial. 6](#_Toc175130956)

# Supplementary Table 1. Baseline / change in efficacy parameters (primary and secondary endpoints) from baseline to week 49 in a Japanese patient and overall population from the COMET trial

|  | **Patient 1 (Japanese) (n=1)** | **Avalglucosidase alfa-arm  (n=51)** |
| --- | --- | --- |
| Upright FVC%, predicted Mean baseline (SD) Mean change from baseline (SE) | 48.04 0.43 | 62.50 (14.4) 2.89 (0.88) |
| 6MWT, m Mean baseline (SD) Mean change from baseline (SE) | 365.00 55.00 | 399.30 (110.9) 32.21 (9.93) |
| 6MWT%, predicted Mean baseline (SD) Mean change from baseline (SE) | 53.13 8.26 | 57.30 (15.00) 5.02 (1.54) |
| MIP%, predicted Mean baseline (SD) Mean change from baseline (SE) | 28.40 0.12 | 51.74 (24.85) 8.70 (2.09)* |
| MEP%, predicted Mean baseline (SD) Mean change from baseline (SE) | 54.23 15.01 | 59.17 (21.60) 10.89 (2.84)* |
| HHD, lower extremity Mean baseline (SD) Mean change from baseline (SE) | 1140 –91.00 | 1330.45 (625.44) 260.69 (46.07) |
| QMFT total score / mean (SE) Mean baseline (SD) Mean change from baseline (SE) | 35 4.00 | 41.29 (10.15) 3.98 (0.63) |
| SF-12 PCS score Mean baseline (SD) Mean change from baseline (SE) | 48.4 4.68 | 35.95 (7.82) 2.37 (0.99) |
| SF-12 MCS score Mean baseline (SD) Mean change from baseline (SE) | 56.2 0.70 | 48.31 (10.11) 2.88 (1.22) |

Abbreviations: 6MWT, 6-min walking test; FVC, forced vital capacity; HHD, hand-held dynamometry; IU, international unit; MCS, mental component summary; MEP, maximal expiratory pressure; MIP, maximal inspiratory pressure; PCS, physical component summary; QMFT, quick motor function test; SD, standard deviation; SE, standard error; SF-12, 12-item short-form health survey.

Note: mean (SD or SE) relate to avalglucosidase alfa-arm (Patient 1 values are actual)

*Two participants with implausibly high MIP% predicted and MEP% predicted values at baseline were excluded from all MIP and MIP analyses.

# Supplementary Table 2. Baseline / changes in efficacy parameters (secondary endpoints) from baseline to week 25 in Japanese patients and overall population from the Mini-COMET trial

|  | **Cohort 1 (20 mg/kg)** | | **Cohort 2 (40 mg/kg)** | |
| --- | --- | --- | --- | --- |
|  | **Patient 2 (Japanese) (n=1)** | **Overall**  **Population**  **(n=6)** | **Patient 3 (Japanese) (n=1)** | **Overall**  **Population**  **(n=5)** |
| GMFM-88 total percent score Mean baseline (SD) Mean change from baseline (SD) | 57.56  2.96 | 54.81 (31.44) 2.62 (9.33) | 96.29 0.83 | 67.43 (33.84) 3.54 (5.46) |
| QMFT total score Mean baseline (SD) Mean change from baseline (SD) | 19 –2 | 23.33 (14.76) −0.17 (4.45) | 52 2 | 31.20 (19.98) 3.20 (4.55) |
| Pompe-PEDI functional skills scale: mobility domain Mean baseline (SD) Mean change from baseline (SD) | 49.83 0.3 | 66.33 (39.46) 8.50 (13.75) | 37.01 0 | 87.20 (48.32) 5.80 (10.87) |
| LVM Z-score Mean baseline (SD) Mean change from baseline (SD) | –1.4 0.6 | –1.10 (1.07) –0.60 (2.16)^a^ | –0.6 –0.1 | 0.13 (2.39) –0.60 (0.71)^b^ |

Abbreviations: GMFM-88, gross motor function measure-88; LVM, left ventricular mass; Pompe-PEDI, Pompe-pediatric evaluation of disability inventory; QMFT, quick motor function test; SD, standard deviation

Note: ^a^n=5, ^b^n=2; mean (SD) relate to the overall populations (Patient 2 and 3 values are actual)

# Supplementary Fig. 1. Course of ADA titer in Patient 1


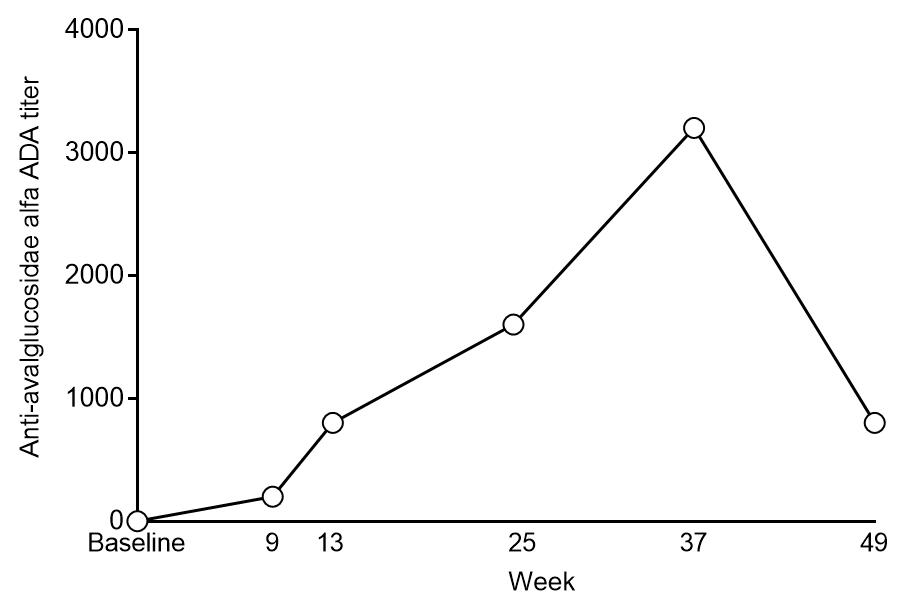


# Supplementary Fig. 2. GMFM-88 total percent score and QMFT total score in (A) Patient 2 (B) Patient 3 (⭘) and the mean (SD) in the overall population in each cohort (⚫) from the Mini-COMET trial.


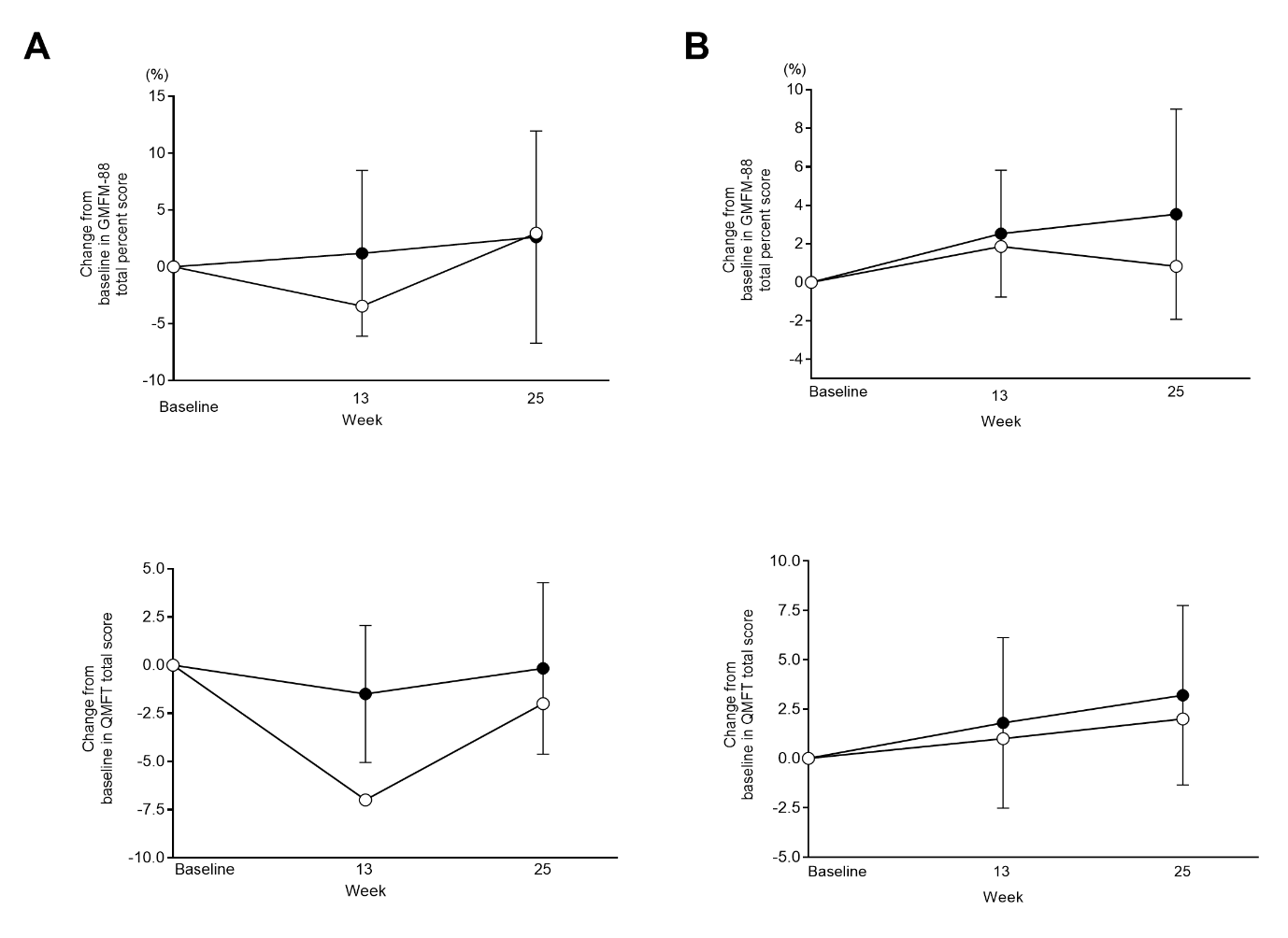

Supplement: Supplementary file 1 — Supplementary material [file mmc1.docx]
